# Anticancer efficacy of biosynthesized silver nanoparticles loaded with recombinant truncated parasporin-2 protein

**DOI:** 10.1038/s41598-024-66650-5

**Published:** 2024-07-05

**Authors:** Monrudee Srisaisap, Panadda Boonserm

**Affiliations:** Institute of Molecular Biosciences, Mahidol University, Phuttamonthon, Salaya, Nakhon Pathom, 73170 Thailand

**Keywords:** Parasporin-2, *Bacillus thuringiensis*, Nanotoxin, Silver nanoparticles, Anticancer effects, Biochemistry, Biological techniques, Cancer

## Abstract

Bacterial toxins have received a great deal of attention in the development of cancer treatments. Parasporin-2 (PS2Aa1 or Mpp46Aa1) is a *Bacillus thuringiensis* parasporal protein that preferentially destroys human cancer cells while not harming normal cells, making it a promising anticancer treatment. With the efficient development and sustainable silver nanoparticles (AgNPs) synthesis technology, the biomedical use of AgNPs has expanded. This study presents the development of a novel nanotoxin composed of biosynthesized silver nanoparticles loaded with the N-terminal truncated PS2Aa1 toxin. MOEAgNPs were synthesized using a biological method, with *Moringa oleifera* leaf extract and maltose serving as reducing and capping agents. The phytochemicals present in *M. oleifera* leaf extract were identified by GC–MS analysis. MOEAgNPs were loaded with N-terminal truncated PS2Aa1 fused with maltose-binding protein (MBP-tPS2) to formulate PS2-MOEAgNPs. The PS2-MOEAgNPs were evaluated for size, stability, toxin loading efficacy, and cytotoxicity. PS2-MOEAgNPs demonstrated dose-dependent cytotoxicity against the T-cell leukemia MOLT-4 and Jurkat cell lines but had little effect on the Hs68 fibroblast or normal cell line. Altogether, the current study provides robust evidence that PS2-MOEAgNPs can efficiently inhibit the proliferation of T-cell leukemia cells, thereby suggesting their potential as an alternative to traditional anticancer treatments.

## Introduction

Cancer is the primary cause of death worldwide, leading to more than 10 million new cases annually^1^. Current cancer therapies, including radiation, chemotherapy, and surgery, are incapable of selectively targeting and can, therefore, damage healthy cells in addition to cancer cells. Moreover, cancers become resistant to chemotherapeutic agents due to repeated chemotherapy treatments^2,3^.

Due to their specificity and cytotoxicity in binding to target cells, bacterial toxin-based therapeutics have recently been suggested as a promising cancer treatment strategy^4–6^. Parasporins, a class of non-insecticidal crystal proteins of *Bacillus thuringiensis* (Bt), are being investigated as prospective anticancer agents due to their specific cytotoxicity to cancer cells^7,8^. So far, six types of parasporins, formerly known as PS1-PS6 or Mpp class for a new nomenclature, have been identified, each exhibiting a specific spectrum and mechanism of action against human cancer cells^9,10^. Parasporin-2Aa1 (PS2Aa1, also known as Cry46Aa1 or Mpp46Aa1 under its new name) is a member of the parasporin family with a β-pore-forming-type structure that has been demonstrated to exhibit a broader cytotoxicity spectrum than others^9,11,12^. PS2Aa1 is synthesized as intracellular parasporal crystals that require solubilization and proteolytic processing to become active against target cancer cells^10^. The processing of PS2Aa1 from a 37-kDa precursor protein by proteinase K digestion at the N and the C termini, results in an active 30-kDa toxin^12^. N-terminal processing of PS2Aa1 by cleaving the first 51 amino acids was found to be essential for cytotoxicity, while C-terminal processing was not essential but did enhance the activity^12^. In its active form, PS2Aa1 is toxic to HepG2, Caco-2, MOLT-4, Jurkat, and HL-60 cell lines but does not exert any toxicity to normal cells^12^. It is believed that the specificity for cancer cells is due to a specific binding to a glycosylphosphatidylinositol (GPI)-anchored protein expressed on cancer cells^12^. Following receptor binding, pore formation causes modifications to the cytoskeletal structures, organelle fragmentation, morphological changes such as cell enlargement, and, ultimately, cell death^12,13^. Although PS2Aa1 is a potential alternative anticancer agent, the delivery of PS2Aa1 free protein to the target cancer cells is hampered by the protein’s inherent limitations, such as its susceptibility to proteolysis, the possible immunogenicity and limited protein uptake by the target cancer cells. The utilization of nanocarriers to transport protein toxins to tumor foci has the potential to enhance specificity, improve intracellular and internalization rates, and minimize systemic toxicity^14^.

Nanoparticles have emerged as powerful tools for biomedical applications due to their unique properties, such as drug delivery, medical imaging, and inherent antibacterial and anticancer activities^15^. Among various nanomaterials, silver nanoparticles (AgNPs) have attracted considerable attention as cancer nanomedicine. The delivery of nanoparticles to cancer cells is achieved either passively through the enhanced permeability and retention (EPR) effect or actively through the presence of different ligands on the surface of nanoparticles, such as antibodies, proteins, and peptides^16^. Anticancer agents or toxins can be actively targeted to cancer cells via AgNPs, allowing for a more specific and efficient cancer therapy method^17^.

Physical, chemical, and biological methods are employed to synthesize silver nanoparticles^18^. It has also been demonstrated that biologically synthesized AgNPs exhibit substantial antitumor activity with fewer adverse effects than those produced by chemical reagents^19,20^. Natural extracts from plants, algae, fungi, and other microorganisms act as reducing and capping agents in biological or green synthesis^21,22^. Plant extracts are superior to microbe-mediated synthesis for the synthesis of AgNPs due to their eco-friendliness, non-pathogenicity, scalability, and reduced cost^21^. Vitamins, proteins, amino acids, enzymes, polysaccharides, alkaloids, and phenolics are among the biomolecules found in plant extracts that function as reducing, capping, and stabilizing agents during the synthesis of AgNPs^21^.

*Moringa oleifera* Lam. (Moringaceae), a common vegetable plant in Africa and Asia, has several bioactive compounds with excellent health benefits, including antioxidant and anticancer properties^23^. *M. oleifera* leaves have been investigated for their anticancer activity^24^. The extract of *M. oleifera* leaves was found to decrease the viability of acute myeloid leukemia, acute lymphoblastic leukemia, and hepatocellular carcinoma cells^25^. Several bioactive compounds found in *M. oleifera* extract, including 4-(α-l-rhamnosyloxy) benzyl isothiocyanate, niazimicin, and β-sitosterol-3-*O*-β-d-glucopyranoside may be responsible for its anticancer properties^26^. It was reported that *M. oleifera* leaf extract could prevent the development of hepatic carcinomas induced with diethyl nitrosamine in rats through the enhancement of antioxidant activity and the induction of apoptosis^27^.

The utilization of nanoparticles loaded with bacterial toxins offers promise as a novel approach to cancer treatment. As previously reported, a nanotoxin composed of truncated pseudomonas exotoxin (PE38) loaded silver nanoparticles (AgNPs) demonstrated a severe cytotoxic effect on the proliferation of breast cancer cells by inducing cell death via the P53-dependent apoptosis mitochondria pathway^28^. Nevertheless, the relatively weak interactions between natural protein toxins and nanoparticles pose a challenge in the production of nanoparticles loaded with protein toxins. Carbohydrates utilized as biomimetic functional molecules on the nanoparticle surface have gained increasing attention among surface coatings^29,30^. Additionally, carbohydrates capped on the surface of nanoparticles can act as a ligand to interact with proteins tagged with carbohydrate-binding molecules such as maltose-binding protein (MBP), hence increasing the loading efficiency of the protein molecules on the nanoparticles.

In this study, the green synthesis of AgNPs was accomplished by utilizing a mixture of silver salt, *M. oleifera* leaf extract, and maltose. This approach employs the capability of *M. oleifera* leaf extract (MOE) as a reducing agent to synthesize silver nanoparticles (MOEAgNPs), which are stabilized by maltose, a reducing disaccharide, as a capping agent. The maltose-capped MOEAgNPs were then loaded with the N-terminally truncated PS2Aa1 fused with MBP (MBP-tPS2) to formulate PS2-MOEAgNPs that were subsequently characterized in terms of size, zeta potential, toxin loading efficacy, and cytotoxic effects on human leukemic T cells compared to normal cells. The study provides evidence that biosynthesized MOEAgNPs can be employed to deliver the parasporin-2 toxin to cancer cells, offering a potential approach for cancer treatment.

## Materials and methods

### Collection and preparation of aqueous extract of *Moringa oleifera* leaves

*Moringa oleifera* leaves were collected from land in Samut Sakhon, Thailand (by Prof. Dr. Panadda Boonserm, Institute of Molecular Biosciences, Mahidol University) and stored for research purposes at the Institute of Molecular Biosciences, Mahidol University, Thailand (13°47′50.5″ north latitudes and 100°19′34.7″ east latitudes) following the guidelines and regulations set by the Department of Agriculture (DOA) under Section 53 of the Plant Varieties Protection Act B.E. 2542 (1999) (Identification number 0539/2566). Fresh *Moringa oleifera* leaves were washed with sterile water to remove dirt. The washed leaves were sun-dried to remove residual moisture and were cut into small pieces. Finely chopped leaves (5 g) were added to 100 mL of boiled sterile water and stirred at 75 °C for 3 h. Then, the mixture was cooled at room temperature and filtered through cheesecloth. The aqueous extract of *M. oleifera* leaves was stored at 4 °C for further use.

### Gas chromatography and mass spectrometry (GC–MS) analysis

GC–MS analysis of MOE was used to determine its phytochemical composition. The GC–MS analysis was conducted at the Mahidol University-Frontier Research Facility (MU-FRF), Thailand, using a GC–MS 7890A-5977B (Agilent Technologies, CA, USA) following the protocol as described previously^31^. The phytochemical compounds from the GC–MS analyses were identified and presented with their compound names, retention times (RT), and molecular formulas using the database of the National Institute of Standards and Technology (NIST17, Gaithersburg, MD, USA).

### Biosynthesis of silver nanoparticles (MOEAgNPs)

The biosynthesis of silver nanoparticles mediated by *M. oleifera* leaf extract and maltose (MOEAgNPs) was performed following the protocol as described previously, with some modifications^31^. A 5 mL dropwise addition of *M. oleifera* leaf extract (MOE) to a 100 mL solution containing a mixture of 1 mM AgNO_3_ and maltose (10 mM or 100 mM) was conducted at room temperature with agitation for about 5 h, or until the solution acquired a colloidal dark brown color indicative of AgNP formation. The mixture was centrifuged at 10,000 rpm for 15 min. The pellet containing AgNPs mediated by MOE and maltose (MOEAgNPs) was rinsed with ultrapure water to eliminate any residual silver ions or leaf extract before use.

### Characterization of MOEAgNPs

#### UV–visible absorbance spectroscopy

The formation of MOEAgNPs was confirmed using UV–Vis spectroscopy with a wavelength range of 190–850 nm (NanoDrop One Microvolume UV–Vis Spectrophotometer, Thermo Fisher Scientific, Waltham, MA, USA).

#### Dynamic light scattering (DLS)

Dynamic light scattering (Horiba SZ-100 particle size analyzer, Kyoto, Japan) was employed to determine the size distribution and surface charge of the MOEAgNPs by injecting them into a disposable cell and a zeta-potential cell equipped with electrodes coated with carbon, respectively.

### Field emission scanning electron microscopy (FE-SEM) coupled with energy dispersive X-ray spectroscopy (EDX), Fourier-transform infrared spectroscopy (FTIR), and X-ray diffraction (XRD) analyses

The size, shape, and basic elements of MOEAgNPs were characterized by using FE-SEM equipped with EDX(JSM-7610FPlus, JEOL, Tokyo, Japan). The sample mixture was centrifuged at 10,000 rpm at room temperature for 15 min. Following multiple washes with ultrapure water, the nanoparticles were collected and air-dried for 48 h. The thin coatings of the sample were deposited onto a carbon-coated copper grid prior to FE-SEM and EDX analyses. The functional groups of MOEAgNPs were identified by using FTIR spectroscopy (Nicolet iS50, Thermo Fisher Scientific Co., Waltham, MA, USA). The pellet sample was prepared using the standard KBr pellet method and analyzed in the transmittance mode in the range of 500–4000 cm^−1^. Crystalline metallic AgNPs were also analyzed by using XRD (Bruker D2 PHASER, Bruker AXS GmbH, Karlsruhe, Germany). The 2Theta was measured from 5° to 90° with a step size of 0.02° using Cu/Kα radiation (*λ* = 1.54184 Å).

### Expression and purification of recombinant MBP-tPS2 protein

*Escherichia coli* BL21(DE3)pLysS cells containing recombinant MBP-tPS2 plasmid were cultured in LB medium with 100 μg/mL ampicillin and 34 μg/mL chloramphenicol. After adding 0.2 mM IPTG to induce protein expression, cells were cultured at 18 °C for 5 h with shaking at 250 rpm. Then, cultured cells were collected by centrifugation at 6000 rpm for 10 min at 4 °C. Cells were then resuspended in an ice-cold Tris buffer (50 mM Tris-HCl pH 8.0, 200 mM NaCl, and 1 mM DTT) and were completely lysed by ultrasonication. The soluble and insoluble fractions were separated by centrifugation at 8,000 rpm at 4 °C for 1 h. The supernatant was loaded into a 5 mL MBPTrap HP column (Cytiva) with Tris buffer. The MBP-tPS2 was eluted by Tris buffer containing 10 mM maltose monohydrate.

### Preparation of MOEAgNPs loaded with MBP-tPS2

Different mass ratios of MOEAgNPs to MBP-tPS2 protein (1:1, 1:2, and 1:3) were prepared with a final volume of 500 μL. The solution was placed on a shaker speed of 300 rpm for 48 h. The solutions were centrifuged at 10,000 rpm for 1 h to determine the protein loading percentage to silver nanoparticles. The supernatant containing unloaded protein was quantified by using gel electrophoresis image analysis software, and loading efficiency was calculated by the below equation:

$${\text{Loading efficiency }}\% \, = \, \left( {{\text{mass of protein used }} - {\text{ protein in the suspension}}/{\text{mass of protein used}}} \right) \, \times { 1}00$$

### In vitro cytotoxicity assay

#### Cell lines and culture conditions

Human foreskin fibroblast cell line Hs68 (ATCC CRL-1635), T-acute lymphoblastic leukemia cells lines: MOLT-4 (ATCC CRL-1582) and Jurkat (ATCC TIB-152 T) were acquired from the American Type Culture Collection (ATCC, Manassas, VA, USA). Hs68 cells were cultivated in Dulbecco’s modified eagle medium (DMEM) (Gibco) supplemented with 10% fetal bovine serum (FBS) (Gibco) and 1% penicillin and streptomycin (Gibco) and maintained at 37 ℃ in a 5% CO_2_ incubator^31^. MOLT-4 and Jurkat cells were cultivated in RPMI-1640 medium (ATCC 30–2001) supplemented with 10% fetal bovine serum (FBS) and 1% penicillin and streptomycin and maintained at 37 °C in a 5% CO_2_ incubator.

### Cell cytotoxicity assay

Cell cytotoxicity was assessed using an MTT (3-(4,5-dimethylthiazol-2-yl)-2,5-diphenyltetrazolium bromide) assay following the protocol as described previously^31,32^. Briefly, Hs68, MOLT-4, and Jurkat cells were grown at about 2 × 10^4^ cells/well in 96 well plates under sterile conditions till approximately 80% confluence. Cells were treated with different concentrations of recombinant MBP-tPS2 protein, MOEAgNPs, and PS2-MOEAgNPs (three replicates for each concentration), and the plate was incubated in a CO_2_ incubator at 37 °C for 24 h. Cell morphological alterations in both types of cells were observed using an inverted light microscope (Nikon Eclipse TS100, Melville, NY, USA). MTT (Invitrogen, Carlsbad, CA, USA) solution (10 μL) was added to each well to a final concentration of 0.5 mg/mL, and the plate was incubated for 4 h. Then, dimethyl sulfoxide (DMSO) (Merck, Darmstadt, Germany) (100 μL) was added to each well to dissolve the formazan crystals, followed by absorbance measurement at 595 nm (Beckman Coulter DTX 880 Microplate Reader). The IC_50_ value was calculated as the test sample concentration required for 50% cell growth inhibition using GraphPad Prism 10.0 (Prism; GraphPad Software, Inc) by fitting the cell viability curve.

### Statistical analysis

All experiments were performed using at least three independent replicates. All data are represented as mean ± SD, and the statistical tests were conducted using GraphPad Prism 10.0 (Prism; GraphPad Software, Inc). Two-way ANOVA was used to compare several groups and a control group. Significance levels are denoted as follows: ns (no significance)* p* > 0.9, * *p* < 0.05, ** *p* < 0.01, *** *p* < 0.001, and **** *p* < 0.0001.

## Results and discussion

### Expression and purification of MBP-tPS2

**Figure 1:**
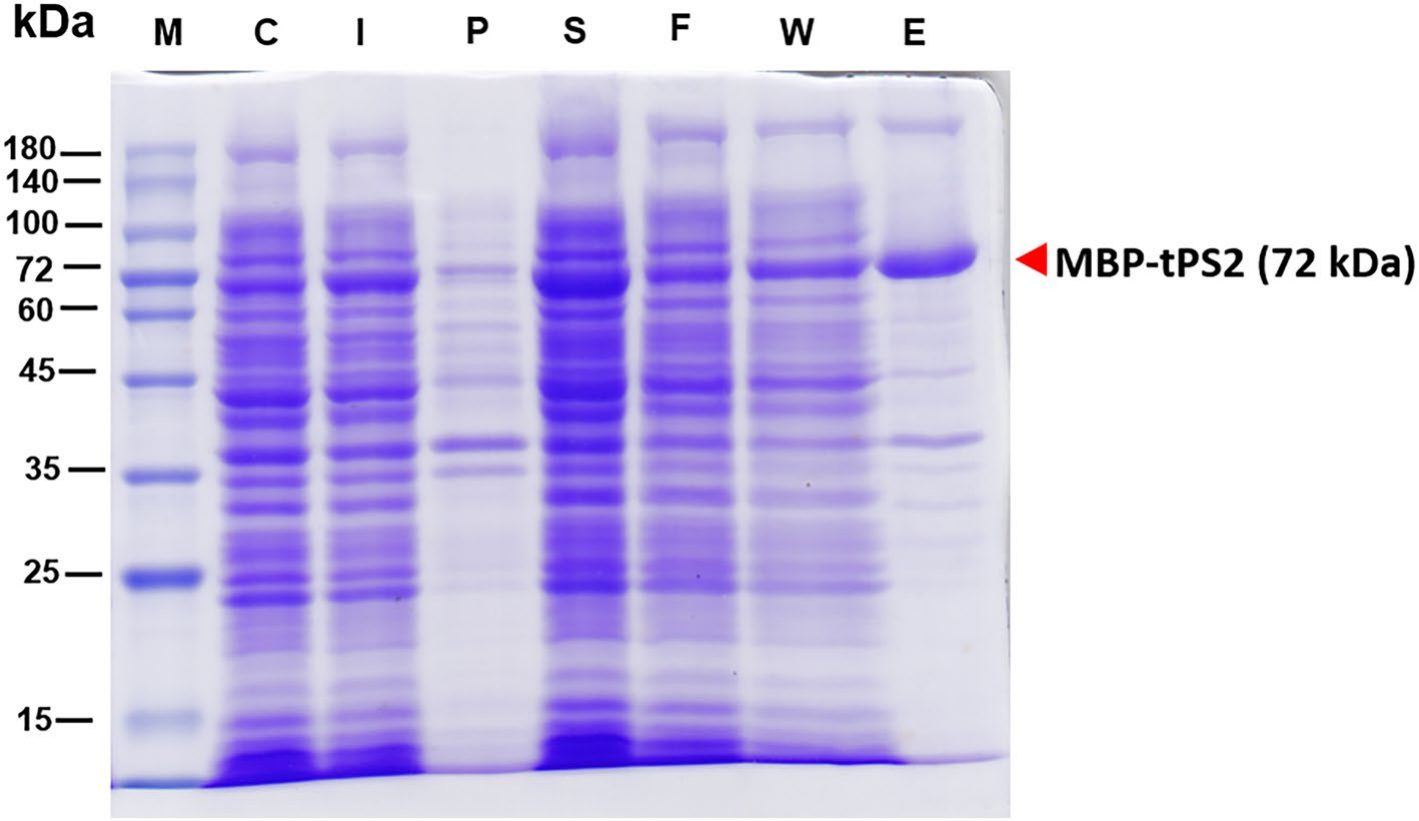
Expression and purification of the MBP-tPS2 fusion protein. Expression of MBP-tPS2 in *E. coli* BL21(DE3)pLysS host cells was induced with 0.2 mM IPTG at 18 °C. The MBP-tPS2 expressed in a soluble form was purified using amylose affinity chromatography. The protein fractions were subjected to 12% SDS-PAGE analysis. M, molecular weight markers; C, total cell proteins before IPTG induction as a negative control; I, total cell proteins after IPTG induction; P, pellet fraction after cell sonication; S, soluble fraction after cell sonication; F, flow through faction; W, wash fraction; E, eluted fraction (with 10 mM maltose) from amylose affinity column. The arrow indicates the molecular mass of the expected MBP-tPS2 protein band.

Our previous study demonstrated that the 37-kDa PS2Aa1 protoxin tagged at its N-terminus with a hexa-histidine sequence was expressed by *E. coli* BL21(DH3)pLysS cells in the form of inclusion bodies. Consequently, inclusion bodies were solubilized using a strong alkaline buffer^33^. Additionally, in order to transform a 37-kDa protoxin into an active 30-kDa form, proteinase K digestion is necessary for the PS2Aa1 protein. For the toxin to exert its cytotoxicity, a minimum of the N-terminal region of PS2Aa1 had to be cleaved, which involved the removal of 51 amino acids^12^. To improve the solubility of the PS2Aa1 protein and obviate the necessity for solubilization and proteinase k activation, we employed maltose-binding protein (MBP) fused to the N-terminal truncated PS2Aa1 as a chaperone, denoted as MBP-tPS2. A soluble form of the recombinant MBP-tPS2 protein with a molecular mass of about 72 kDa was successfully produced in *E. coli* in combination with a low induction temperature of 18 °C. Purification of MBP-PS2 fused proteins was conducted using amylose affinity chromatography (Fig. 1). After endotoxin removal, the endotoxin level of the purified protein was 0.42 EU/μg, as measured by the Limulus amebocyte lysate (LAL) endotoxin assay which is lower than the maximum allowed for biological products ( < 1 EU/μg)^34^.

### Gas chromatography and mass spectrometry (GC–MS) analysis of an aqueous *Moringaoleifera* leaf extract (MOE)

An aqueous *M. oleifera* leaf extract (MOE) was analyzed using GC–MS to identify bioactive compounds that may have acted as a reducing agent for free metal cations (Ag^+^ to Ag^0^), a stabilizing agent of AgNPs during the nucleation phase and a capping agent for fully grown or stabilized AgNPs (Table 1 ). Four major volatile organic compounds (VOCs), which are known as specialized metabolites, were identified from MOE, including phenols, β-Ionone, benzoyl isothiocyanate, and benzofuranone. The medicinal properties exhibited by the extracts of *M. oleifera* have been attributed to these bioactive compounds^35,36^. A principal role of phenolic compounds is to reduce oxidative stress by scavenging free radicals, thereby preventing disorders associated with oxidative stress^37^.

**Table 1:** The major bioactive compounds of *M. oleifera* leaf extract (MOE) analyzed by GC–MS.

No	RT (min)	Compound name	Formula
1	6.947	Benzoyl isothiocyanate	C_8_H_5_NOS
2	24.288	3-hydroxy-β-ionone	C_13_H_20_O
3	25.072	Phenol, 3,5-bis(1,1-dimethylethyl)	C_14_H_22_O
4	25.539	2(4*H*)-Benzofuranone, 5,6,7,7a tetrahydro-4,4,7a-trimethyl	C_11_H_16_O_2_

One of the major VOCs identified from MOE is β-Ionone. β-Ionone is a natural plant volatile compound that has received increased attention for its potential as an anticancer agent and for other human health benefits^38^. Anticancer activities of β-ionone have been demonstrated in melanoma, breast cancer, and chemical-induced rat carcinogenesis^39^. A previous report revealed that MOE containing the 3-hydroxy- β-ionone active compound strongly inhibited cell proliferation and induced apoptosis of the human squamous cell carcinoma 15 (SCC15) cell line^40^.

Another major active constituent of MOE discovered in this study is benzoyl isothiocyanate. Some evidence suggests that the leaves and seeds of *M. oleifera* are abundant in an isothiocyanate compound with anticancer, hypolipidemic, anti-inflammatory, and antioxidant properties^41,42^. Isothiocyanate has been shown to possess anticancer properties in several studies, including hepatocellular carcinoma Hep3B cells^43^, colon cancer Caco-2 cells^44^, human prostate cancer PC-3 cells^45^, and SH-SY5Y human neuroblastoma cells by regulating NF-κB and apoptosis-related factors^46^.

Benzofuranone is a benzofuran derivative identified as one of the significant phytochemical compounds in MOE. Potential anticancer activity was demonstrated by benzofuran derivatives, which also exhibited a reduced incidence or severity of adverse effects that are typically associated with chemotherapeutic treatments^47^.

### Biological synthesis and characterization of MOEAgNPs and PS2-MOEAgNPs

**Figure 2:**
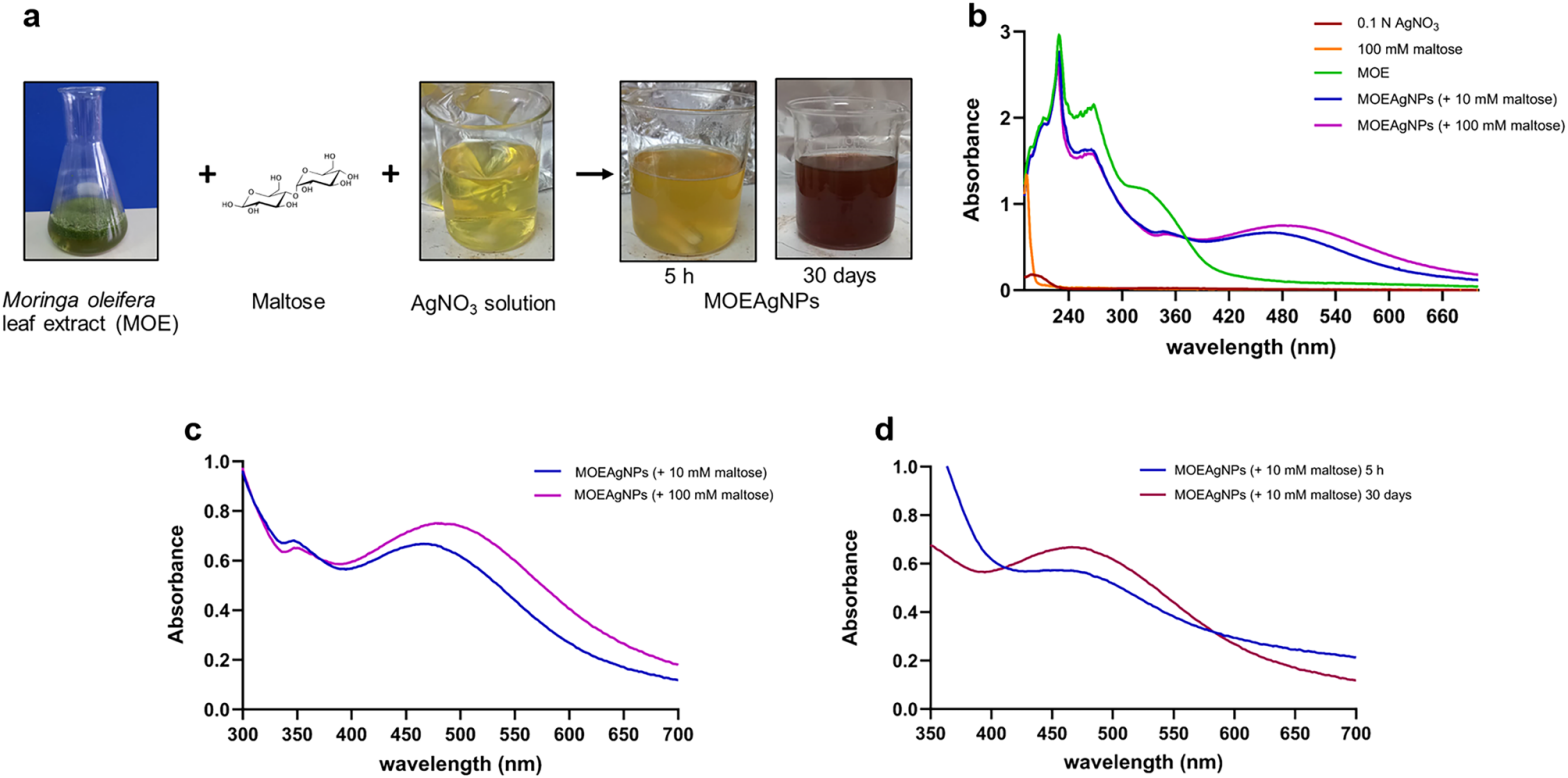
Characterization of AgNPs using *M. oleifera* leaf extract and maltose (MOEAgNPs). Changing color of the reaction mixture after 5 h and 30 days of incubation (**a**) UV–Vis spectra of MOEAgNPs compared to silver nitrate solution (AgNO_3_), maltose, or MOE alone (**b**). The UV–Vis spectra reveal the SPR peaks with different maltose concentrations (**c**) and incubation times (**d**).

The reaction mixture of silver nitrate solution (AgNO_3_), *M. oleifera* leaf extract (MOE), and maltose exhibited a dark yellow color within approximately 5 h, indicating the formation of AgNPs mediated by MOE (MOEAgNPs) (Fig. 2a). The alteration in color observed can be attributed to the excitation of surface plasmons by the silver nanoparticles and the change of Ag^+^ ions to metallic Ag^0^ by reducing agents^48^. This was further validated by observing a prominent absorption band at around 450 nm (Fig. 2b), which corresponds to the SPR band of AgNPs in the range of 350–500 nm. There was no apparent evidence of this absorption observed in the AgNO_3_ solution, the aqueous extract of *M. oleifera* leaves, or maltose alone (Fig. 2b). Maltose served two roles in our biosynthesis: it facilitated the reduction of Ag^+^ ions to Ag^0^ atoms and acted as a capping agent to stabilize the silver nanoparticles. As the concentration of maltose in the reaction mixture increased from 10 to 100 mM, a red shift in the absorbance band to a longer wavelength (from 450 to 480 nm) was observed, as shown in Fig. 2c, indicating that particle size increases with increasing maltose concentration. Thus, a reduced maltose (10 mM) concentration is more suitable for the biosynthesis of MOEAgNPs. Furthermore, the colloid solution was kept at 4 °C for approximately one month. Over time, as shown in Fig. 2a, the color intensity of the colloid solution gradually increased from dark yellow to dark brown, together with the increased absorption intensity of the SPR band (Fig. 2d), indicating a greater extent of Ag^+^ reduction and a greater quantity of MOEAgNPs. The formation of MOEAgNPs was further confirmed by Dynamic light scattering, Field Emission Scanning Electron Microscopy (FE-SEM) Coupled with Energy Dispersive X-ray Spectroscopy (EDX), Fourier-Transform Infrared Spectroscopy (FTIR), and X-ray Diffraction (XRD) Analyses.

**Figure 3:**
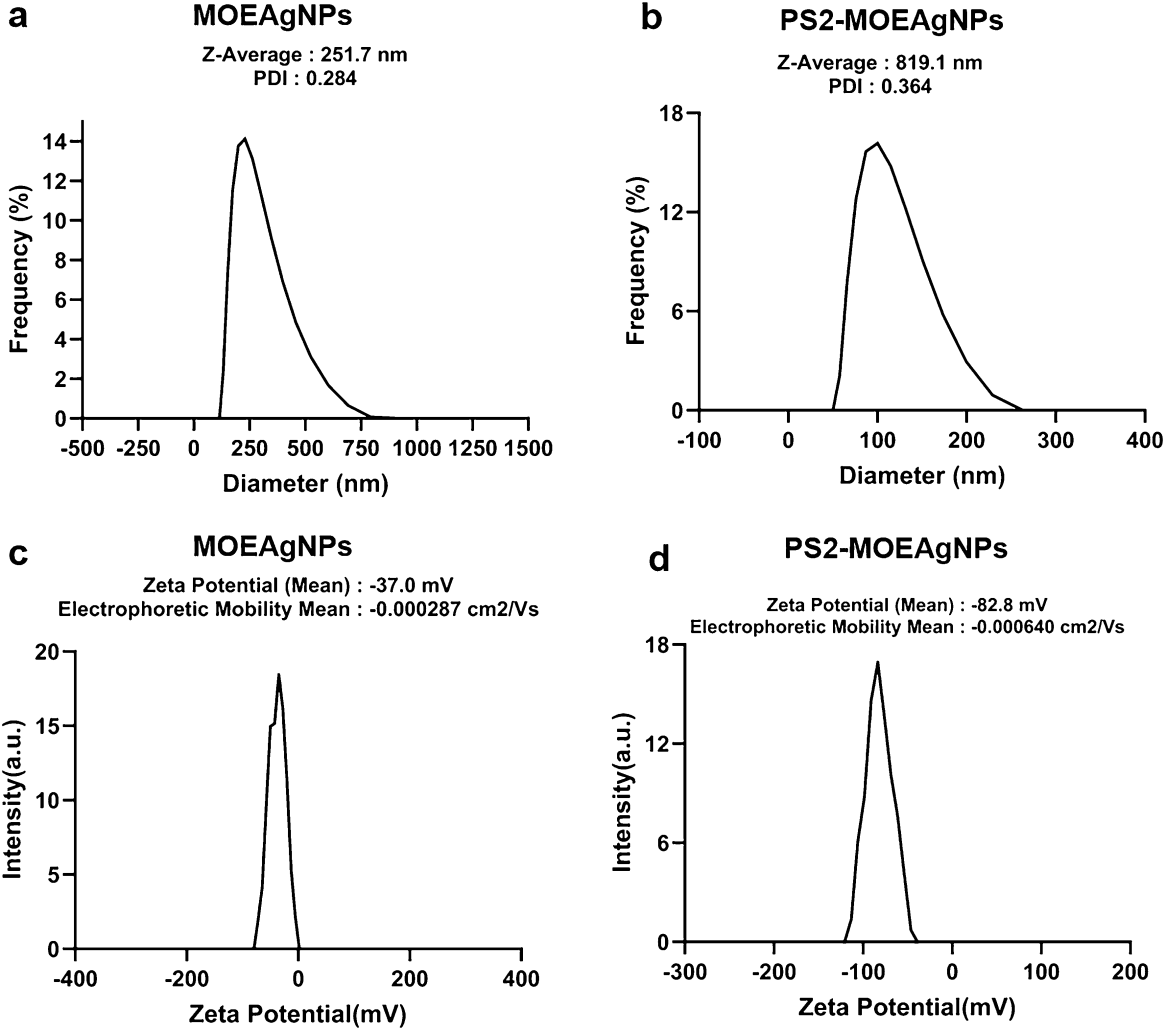
Dynamic light scattering (DLS) analysis of MOEAgNPs (**a**) and PS2-MOEAgNPs (**b**); zeta potential analysis of MOEAgNPs (**c**) and PS2-MOEAgNPs (**d**).

The biosynthesized MOEAgNPs surface charge and hydrodynamic size distribution were determined using dynamic light scattering (DLS). As determined by dynamic light scattering (DLS), the average particle size of MOEAgNPs was around 250 nm with a polydispersity index (PDI) of 0.28 (Fig. 3a). PDI indicates the quality of size distribution of a nanoparticle and the values around 0.3 are optimal^49^. The zeta potential, which characterizes the stability of nanoparticles in aqueous solutions and their surface charge potential, was determined to be around − 37 mV using DLS analysis (Fig. 3c). Nanoparticles with zeta potentials larger than +30 mV or less than −30 mV are referred to as strongly cationic or strongly anionic, respectively, which prevents nanoparticle aggregation^50^.

**Figure 4:**
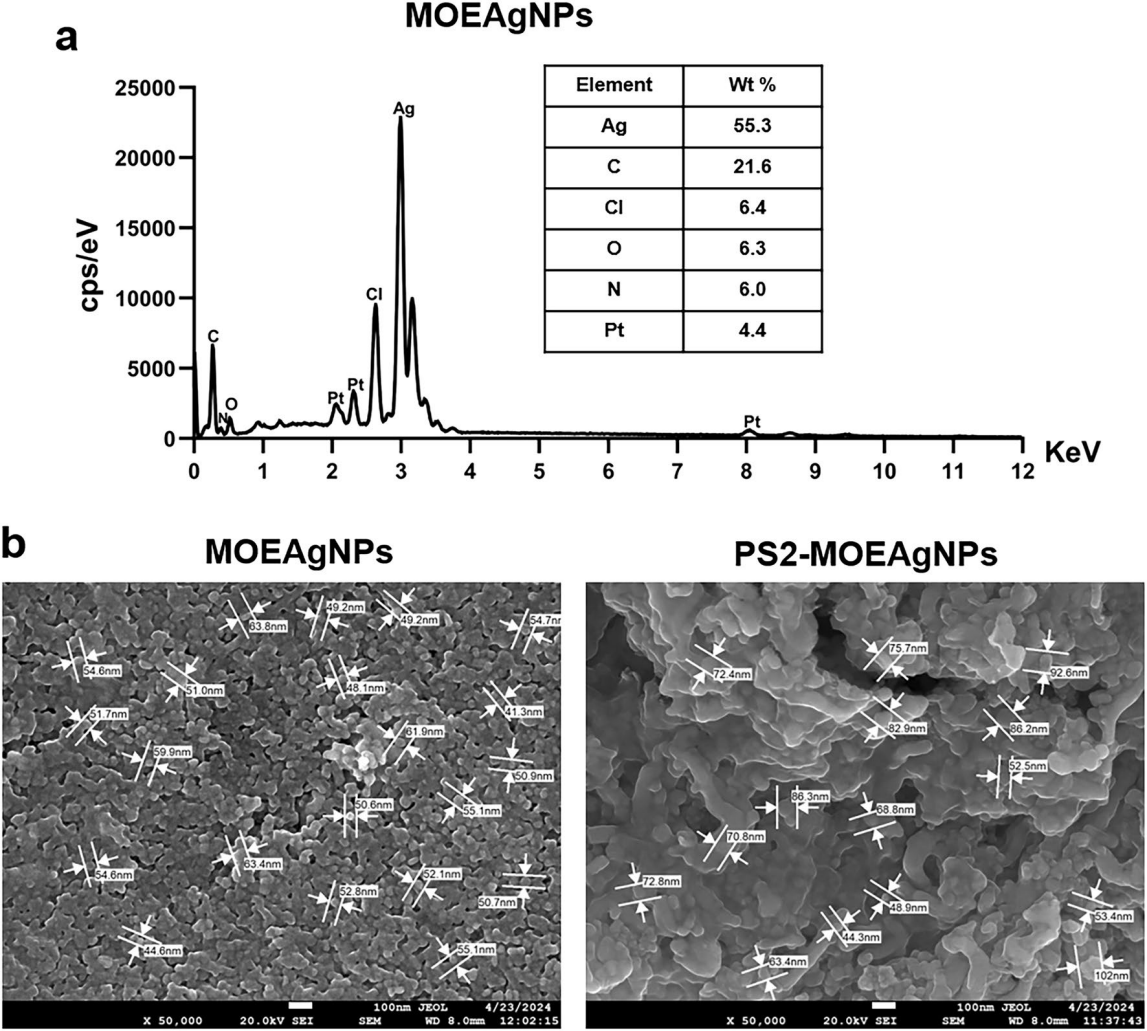
EDX analysis of MOEAgNPs (**a**) FE-SEM images of MOEAgNPs and PS2-MOEAgNPs (**b**).

The EDX spectrum confirmed the presence of silver in MOEAgNPs, which exhibited a prominent characteristic peak at 3 keV^51^, indicating that silver constituted almost 60 % of the elements (Fig. 4a). Weak signals from C and O were detected in addition to the significant peak of Ag, most likely due to phytochemicals and maltose capping AgNPs.

The size and morphology of MOEAgNP were determined by an FE-SEM equipped with an EDX detector. The acquired micrographs (50,000×) showed uniform and spherical shapes of MOEAgNPs, with their sizes ranging from 50.0 to 60.0 nm (Fig. 4b), which are much smaller than the average particle size obtained from DLS analysis, most likely because the DLS technique measures the diffusion coefficient of suspended nanoparticles undergoing Brownian motion in a solution by analyzing the fluctuating scattered intensity of the nanoparticles in the solution rather than their physical size^52^.

The particle size and zeta potential of MOEAgNPs after loading with recombinant MBP-tPS2 protein, hereafter referred to as PS2-MOEAgNPs, were determined by DLS. Protein adsorption on the nanoparticle surface often results in an increase in the nanoparticles’ hydrodynamic size. Results showed a substantial change in the hydrodynamic radius of PS2-MOEAgNPs ( ~ 800 nm) compared with that of the MOEAgNPs (Fig. 3b), and the FESEM image analysis further confirmed the larger particle size of PS2-MOEAgNPs (~70–100 nm) compared with that of the MOEAgNPs (Fig. 4b). The observed increase in particle size confirmed the presence of a protein corona surrounding MOEAgNPs after 48 h of incubation with the MBP-tPS2 protein. This protein corona likely promoted the agglomeration induced by the adsorbed protein layer, which became more noticeable with extended incubation periods. In this study, the maltose-capped surface of MOEAgNPs was designed to act as a ligand to bind with MBP-tPS2 via maltose-MBP interaction, hence enhancing the loading efficiency of protein molecules on the nanoparticles. However, the interaction between proteins and nanoparticle surfaces is complicated by the presence of hydrophilic and hydrophobic interactions, necessitating detailed information for a thorough understanding of the phenomena in the physiological environment and successful therapeutic use.

The attachment of MBP-tPS2 on MOEAgNPs was also confirmed by measuring the surface charge, which changed from − 37 mV (MOEAgNPs) to − 83 mV (PS2-MOEAgNPs) (Fig. 3d). Previous study has indicated that the surface composition of MBP, which exhibits a negative charge at neutral pH (pI 5.22), comprises approximately 28 % negatively charged residues and 28 % positively charged residues^53^. In addition, PS2Aa1 protein has an isoelectric point within the acidic range of 5.12–6.19^54^. The PS2-MOEAgNPs have a higher negative zeta potential than MOEAgNPs, indicating that the MBP-tPS2 protein has a negative charge at the neutral pH of the reaction mixture. The stability of PS2-MOEAgNPs is also enhanced by the increase in negative charge, which prevents sample aggregation as a result of the repulsion force between adjacent negatively charged particles.

### FTIR and XRD analyses of MOEAgNPs

**Figure 5:**
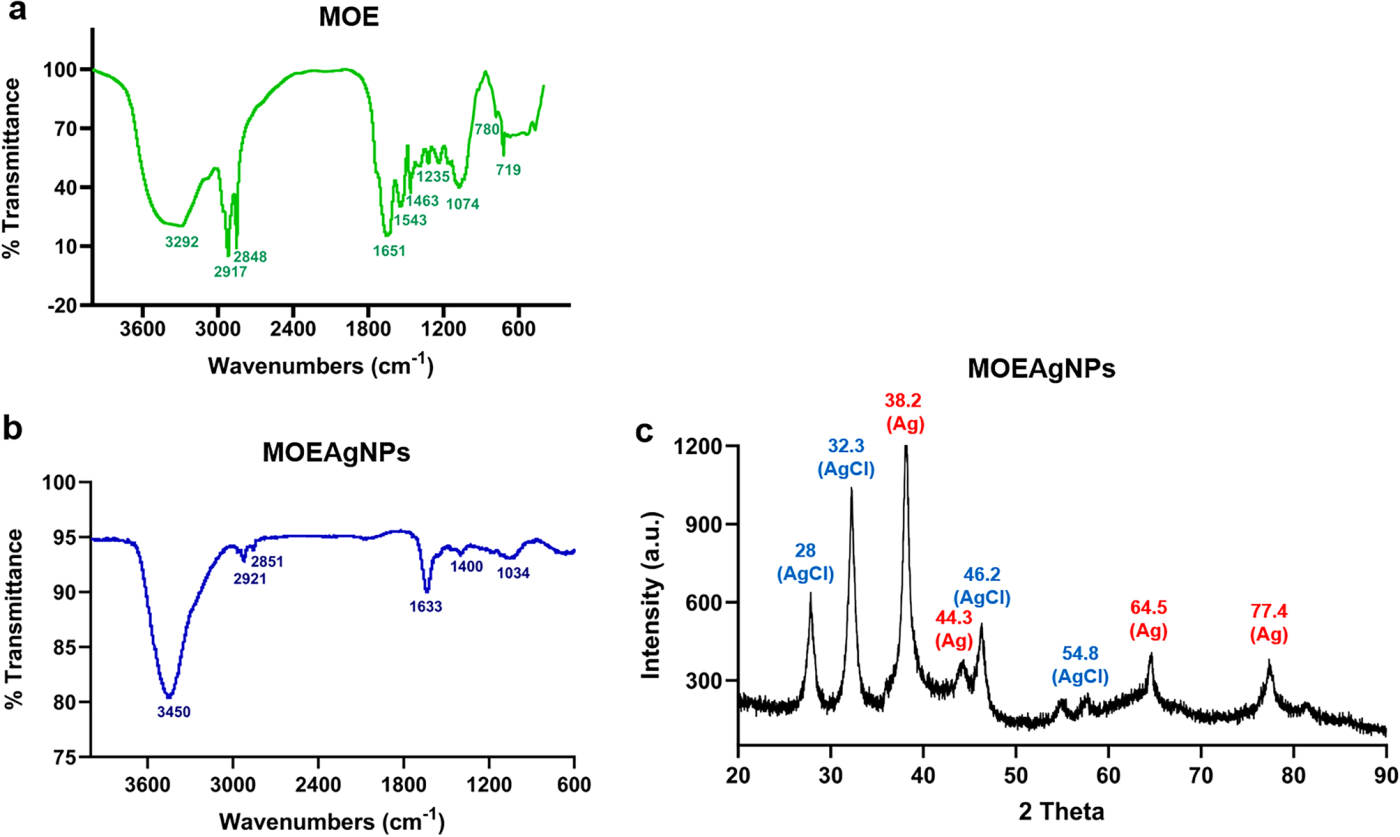
FTIR spectra of MOE (**a**) and MOEAgNPs (**b**) and XRD spectra of biosynthesized MOEAgNPs (**c**).

An analysis of the functional groups present in MOEAgNPs in comparison to MOE was conducted using FTIR spectra, which are illustrated in Fig. 5a and b. FTIR results demonstrated the transmittance of MOE at 3292, 2917, 2848, 1651, 1543, 1463, 1235, 1074, 780, and 719 cm^−1^ (Fig. 5a), whereas MOEAgNPs displayed spectral peaks at 3450, 2921, 2851, 1633, 1400, and 1034 cm^−1^ (Fig. 5b). MOEAgNPs exhibited a strong shift to the higher frequency at 3450 cm^−1^ (Fig. 5b), suggesting that the OH groups present in phenolic compounds of MOE and maltose facilitate the reduction of silver ions to silver nanoparticles and potentially function as a stabilizing or capping agent on MOEAgNPs, in contrast to the broad peak observed in the vicinity of 3292 cm^−1^ in MOE (Fig. 5a), which corresponds to the OH stretching prevalent in phenolic compounds. Furthermore, a slight shift was observed in the MOEAgNPs spectrum near 1633 cm^−1^, which corresponds to carbonyl stretching (Fig. 5b). This indicates that carbonyl groups present in MOE and maltose are implicated in the bio-reduction and capping of MOEAgNPs.

In order to validate the crystalline characteristics of MOEAgNPs, an XRD pattern was collected (Fig. 5c). The XRD analysis revealed four significant peaks at 2θ values: 38.20° (111), 44.30° (200), 64.50° (220), and 77.41° (311) (Fig. 5c). These peaks correspond to the crystallographic planes of the face-centered cubic (*fcc*) of silver crystal, as reported on card No. 00-004-0783 of the Joint Committee on Power Diffraction Standards (JCPDS)^55^. These results indicate that the MOE-mediated AgNPs in this study possess a nanocrystalline structure. In addition, the (111), (200), (220), and (311) planes of the AgCl-NPs (JCPDS card No. 31–1238) were identified as the origins of the peaks observed at 2 θ values of 28°, 32.3°, 46.2°, and 54.8°, respectively, as reported previously^56^ (Fig. 5c). Ag^+^ ions from AgNO_3_ and Cl^−^ ions from phytochemical compounds in MOE may react to generate AgCl-NPs.

### Toxin loading efficiency

In order to optimize the loading efficiency of MBP-tPS2 onto the biosynthesized MOEAgNPs, we designed a methodology in which MOEAgNPs were coated with maltose, which functions as a capping ligand for the binding of MBP-tPS2 through maltose-MBP interaction. By varying the mass ratios of MOEAgNPs to MBP-tPS2, we found that the 1:3 mass ratio provided the highest loading efficiency, accounting for around 60%, while 1:1 and 1:2 mass ratios yielded approximately 26% and 42%, respectively. UV–vis spectroscopy was also employed to evaluate the protein’s absorption spectrum, which corresponds to the absorption spectrum at around 280 nm found in the PS2-MOEAgNPs sample. The absorption peaks at around 280 nm were observed in PS2-MOEAgNPs at various mass ratios of MOEAgNPs to MBP-tPS2, with the 1:3 mass ratio giving the highest absorption intensity of the 280 nm peak (see Supplementary Fig. S1), which corresponds to the highest loading efficiency of MBP-tPS2 onto the biosynthesized MOEAgNPs.

### MBP-tPS2, MOEAgNPs, and PS2-MOEAgNPs exhibited anticancer activity against human leukemic T cells

**Figure 6:**
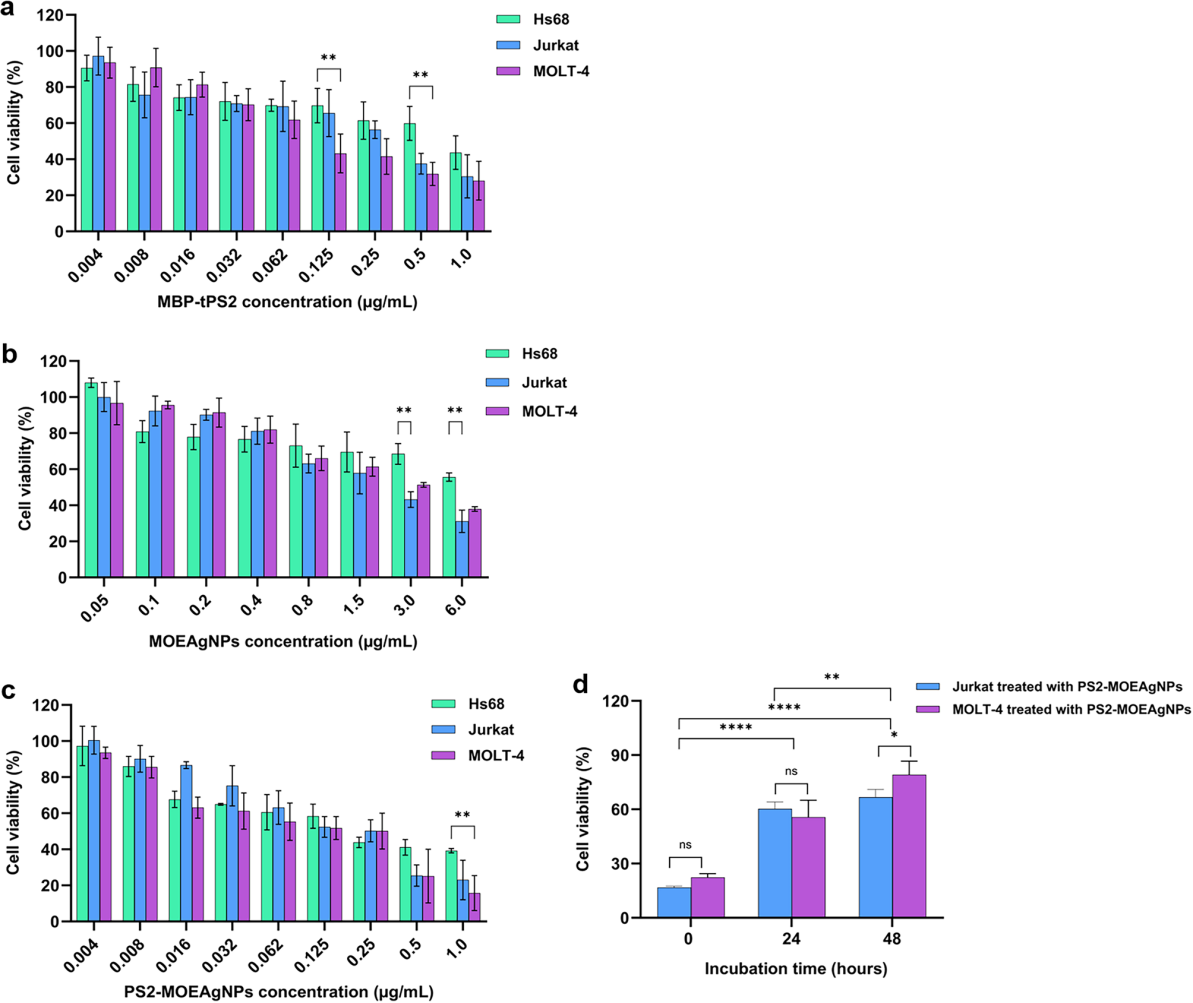
Percentages of cell viability of Hs68, Jurkat, and MOLT-4 cell lines in response to MBP-tPS2, MOEAgNPs, and PS2-MOEAgNPs treatments and functional stability of PS2-MOEAgNPs. After 24 h of incubation with various concentrations of MBP-tPS2 **a**, MOEAgNPs **b**, and PS2-MOEAgNPs **c**, the percentage of cell viability was determined by MTT assay by comparing the absorbance of each sample to that of the negative control (PBS, pH 7.4). Functional stability of PS2-MOEAgNPs was assessed by measuring the anti-proliferative activity against Jurkat and MOLT-4 cells after pre-incubating PS2-MOEAgNPs (1 μg/mL) at 37 °C for 24 and 48 h compared with the unincubated sample **d**. All data are presented as mean ± SD from the triplicate analyses. Statistical significance was analyzed using two-way ANOVA and annotated as follows: **** *p* < 0.0001, **p < 0.01, *p < 0.05, and ns (no significance)* p* > 0.9.

It has been reported that PS2Aa1, in its active form, exhibits cytotoxicity towards the HepG2, Caco-2, MOLT-4, Jurkat, PC-3, MCF-7, and HL-60 cancer cell lines, while normal cells remain unaffected^12,13,32^. In order to assess the cytotoxic properties of recombinant MBP-tPS2 protein, this study examined the effects on human leukemic T cells (Jurkat and MOLT-4) and normal human skin-fibroblast cells (Hs68) when exposed to various concentrations of the protein for 24 h. Based on the MTT assay, the recombinant MBP-tPS2 protein induced cytotoxicity in Jurkat and MOLT-4 cells by inhibiting their proliferation in a dose-dependent manner, with their half-maximal inhibitory concentrations (IC_50_) values of 0.04 μg/mL and 0.03 μg/mL, respectively (Fig. 6a, Table 2). However, normal Hs68 cells were only affected when exposed to high concentrations of the MBP-tPS2 protein (above 0.5 μg/mL), with an IC_50_ value of > 1.0 μg/mL (Fig. 6a, Table 2). In addition, we assessed the cytotoxicity of MBP alone on both cancerous and non-cancerous cell lines. We found no evidence of cytotoxicity against both cancer and normal cells with MBP alone (data not shown), suggesting that the cytotoxicity observed in cancer cells is due to the N-terminal truncated form of PS2Aa1. Our findings indicated that the recombinant MBP-tPS2 protein had the potential to preferentially destroy cancer cells.

**Table 2:** IC_50_ (µg/mL) values of Hs68, Jurkat, and MOLT-4 cell lines after treatments with MBP-tPS2, MOEAgNPs, and PS2-MOEAgNPs.

Treatment	IC_50_ (μg/mL) IC_50_ (μg/mL)		
	Hs68	Jurkat	MOLT-4
MBP-tPS2	> 1.0	0.04	0.03
MOEAgNPs	> 6.0	1.80	2.20
PS2-MOEAgNPs	> 0.5	0.07	0.02

MOEAgNPs at various concentrations were tested for their anti-proliferative effects on the Jurkat and MOLT-4 cancer cell lines, as well as the Hs68 fibroblast cell line. MOEAgNPs-treated Jurkat and MOLT-4 cells displayed dose-dependent inhibition of cell growth, with IC_50_ values of 1.80 μg/mL and 2.20 μg/mL, respectively, but Hs68 cells exposed to MOEAgNPs had fewer cytotoxic effects, with an IC_50_ value of > 6.0 μg/mL (Fig. 6b, Table 2). MOEAgNPs potentially exhibited anticancer properties due to the presence of phenols and other volatile phenolic compounds, including β-Ionone, benzoyl isothiocyanate, and benzofuranone, as identified in this study (Table 1). Consistent with our findings, it has been demonstrated that AgNPs are harmless to human cells at low concentrations^31,57^. The observed cytotoxic effects on Hs68 normal cells in response to MOEAgNPs at higher concentrations indicate that non-cancerous cells may potentially uptake the silver nanoparticles. However, the cellular uptake efficiency of non-cancerous cells remains inferior to that of cancer cells, resulting in lower cytotoxicity than that observed in cancer cells. This observation is consistent with previous studies that have demonstrated the cytotoxic effects of silver nanoparticles on normal cell lines, including human embryonic kidney cells and fibroblast-derived cells^58,59^.

The nanoparticle surface can modify the structure of the protein loaded onto it, thus affecting the protein’s overall biological activity. Although we did not directly assess the conformational change of MBP-tPS2 after absorption with the nanoparticles, we did employ the cytotoxicity assay to indirectly validate the protein’s biological activity after loading it onto biosynthesized MOEAgNPs. As demonstrated in Fig. 6c, PS2-MOEAgNPs inhibited Jurkat and MOLT-4 cell proliferation in a dose-dependent manner, with IC_50_ values of 0.07 μg/mL and 0.02 μg/mL, respectively. In contrast, PS2-MOEAgNPs inhibited the proliferation of Hs68 cells by 15–40% at concentrations ranging from 0.008 to 0.25 µg/mL, and the effect remained steady as the concentrations increased to 0.5 and 1.0 µg/mL. As a result, the cell viability curve fitting was marginally biased. Consequently, the IC_50_ value was reported to be greater than 0.5 µg/mL for HS68 cells treated with PS2-MOEAgNPs. In addition, a comparative analysis of the cytotoxic effects of PS2-MOEAgNPs and MOEAgNPs at all concentrations of silver nanoparticles on the Jurkat and MOLT-4 cancer cell lines revealed that the former exhibited more pronounced effects. The IC_50_ values of PS2-MOEAgNPs against MOLT-4 (0.02 μg/mL) and Jurkat (0.07 μg/mL) cells were much smaller than those of MOEAgNPs against MOLT-4 (2.20 μg/mL) and Jurkat (1.80 μg/mL) cells (Table 2). These findings imply that the cytotoxic efficacy of MOEAgNPs was enhanced by the loading of MBP-tPS2 onto them due to the combined cytotoxic effects induced by both MOEAgNPs and MBP-tPS2. The enhanced cytotoxicity of PS2-MOEAgNPs also indicates that the protein conformation and function of MBP-tPS2 have not been disrupted due to protein-NP or protein–protein interactions, even though the MBP-tPS2 likely forms a protein corona that contributes to the significantly larger size of the PS2-MOEAgNPs in comparison to the MOEAgNPs.

The pathological and cell death processes were evaluated by comparing the morphological alterations of the cells treated with the PS2-MOEAgNPs to those of the MBP-tPS2 (free protein) and MOEAgNPs (free silver nanoparticles). When treated with PS2-MOEAgNPs, Jurkat and MOLT-4 cells exhibited similar dose-dependent morphological alterations, including intracellular vacuolations and nuclear shrinkage, which are features of cell apoptosis, and subsequent cell lysis (see Supplementary Fig. S2). In contrast, when treated with PS2-MOEAgNPs, MBP-tPS2, or MOEAgNPs, Hs68 cells showed significantly fewer morphological changes, including some small vacuolated structures, a lower level of elongated cells (typical fibroblast cell morphology), and the presence of some rounded cells when compared to the control group (see Supplementary Fig. S2). The fact that PS2-MOEAgNPs caused significant changes in cell morphology comparable to those seen with MBP-tPS2 suggests that MBP-tPS2, after loading onto MOEAgNPs, could still interact with the cell membrane, resulting in the formation of pores that lead to cell death via late apoptosis or necrosis. This is consistent with our previous findings that PS2Aa1 caused cell necrosis and lysis in HK1 and HepG2 cells^32,60^, implying that necrosis may be the cell death mechanism of PS2Aa1. In addition, the activation of caspases and the regulation of survival/death pathways, such as AKTs, have been demonstrated to be involved in cell death through the induction of apoptosis in the PS2 protein^13^. Several mechanisms for the anticancer effects of AgNPs have been proposed. AgNPs can trigger apoptosis or necrosis by damaging the ultrastructure of cancer cells, inducing ROS production and DNA damage, inactivating enzymes, and regulating signaling pathways^61^. Therefore, the enhanced cytotoxicity of PS2-MOEAgNPs is expected to generate more apoptotic or necrotic effects in treated cells than MOEAgNPs and MBP-tPS2. In addition, the ability of MOEAgNPs to enter target cells as nanocarriers and transport MBP-tPS2 with them may account for the increased anticancer efficacy of PS2-MOEAgNPs.

### Stability of PS2-MOEAgNPs upon incubation at 37 °C and 4 °C

The functional stability of PS2-MOEAgNPs was assessed by measuring the residual anti-proliferative activity against Jurkat and MOLT-4 cells after pre-incubating PS2-MOEAgNPs (1 μg/mL) at 37 °C for 24 and 48 h compared with the unincubated sample. PS2-MOEAgNPs at 1 μg/mL reduced cytotoxic activity against Jurkat and MOLT-4 cells by 40% after 24 h of incubation at 37 °C and 50–60% after 48 h (Fig. 6d). In addition, the PS2-MOEAgNPs were stored at 4 °C for four days before being treated with the leukemic T cells. The Jurkat and MOLT-4 cells’ morphological changes and MTT assays revealed that the cytotoxicity of the PS2-MOEAgNPs remained unaltered after four days of storage at 4 °C (data not shown). Nevertheless, further study is required to evaluate the stability of the PS2-MOEAgNPs at different storage conditions and their shelf life.

## Conclusion

This is the first study to examine the anticancer effects of biosynthesized AgNPs mediated by *M. oleifera* leaf extract and maltose, as well as a novel nanotoxin composed of biosynthesized AgNPs loaded with the N-terminal truncated PS2Aa1 toxin fused with maltose-binding protein (PS2-MOEAgNPs). PS2-MOEAgNPs exhibit superior anticancer activity against human leukemic T cells compared to biosynthesized MOEAgNPs. This may be attributed to the additive cytotoxic effects induced by both MOEAgNPs and MBP-tPS2. The primary goal of cancer treatment is to identify anticancer agents with a high ability to selectively induce cancer cell death. In this investigation, we provide preliminary evidence that PS2-MOEAgNPs may cause both late apoptosis and necrosis in human leukemic T cells with less effect on normal cells. However, more investigation is needed to determine the underlying molecular mechanism of cell death induced by PS2-MOEAgNPs. In addition to in vitro investigation, a series of in vivo tests in a real physiological setting is needed to assess the biodistribution, targeted delivery, and potential toxicity of PS2-MOEAgNPs for future therapeutic applications.

### Supplementary Information

Supplementary Figures.

## Data Availability

All data are available from the corresponding author upon request.
