# Supplementary material for: Anticancer efficacy of biosynthesized silver nanoparticles loaded with recombinant truncated parasporin-2 protein
Source: Sci Rep. 2024 Jul 5;14:15544. doi: 10.1038/s41598-024-66650-5 (PMC11226667; doi:10.1038/s41598-024-66650-5)
Supplement: Supplementary file 1 — Supplementary Figures. [file 41598_2024_66650_MOESM1_ESM.pdf]

Anticancer efficacy of biosynthesized silver nanoparticles loaded with recombinant truncated parasporin-2 protein

Monrudee Srisaisap[1], Panadda Boonserm[1*]

[1]Institute of Molecular Biosciences, Mahidol University, Salaya, Phuttamonthon, Nakhon Pathom, Thailand 73170

*Corresponding author:

Panadda Boonserm Tel.: (66) 2441-9003; Fax: (66) 2441-9906;

E-mail: panadda.boo@mahidol.ac.th

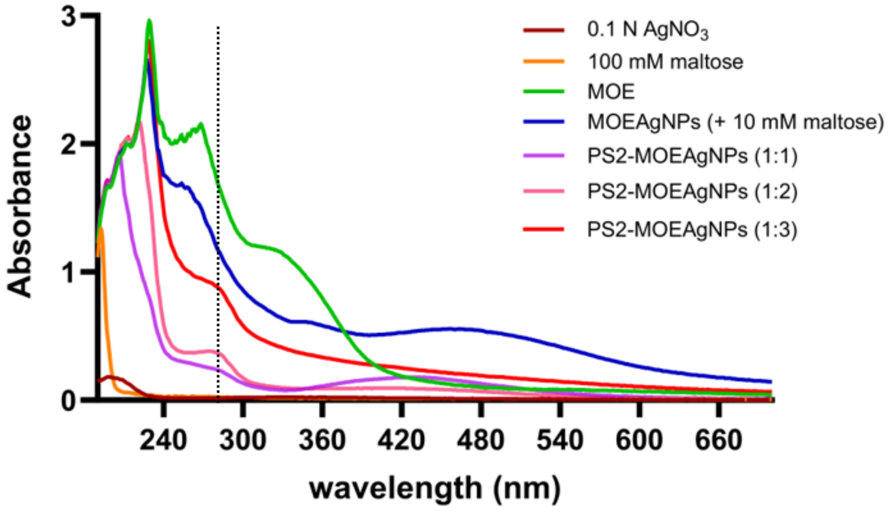

Supplementary Figure S1. UV-Vis spectra of PS2-MOEAgNPs at various mass ratios of MOEAgNPs to MBP-tPS2 (1:1, 1:2, and 1:3) compared to MOEAgNPs, silver nitrate solution ($AgNO_3$), 100 mM maltose, or MOE alone. The dashed line indicates the absorption peaks at 280 nm.

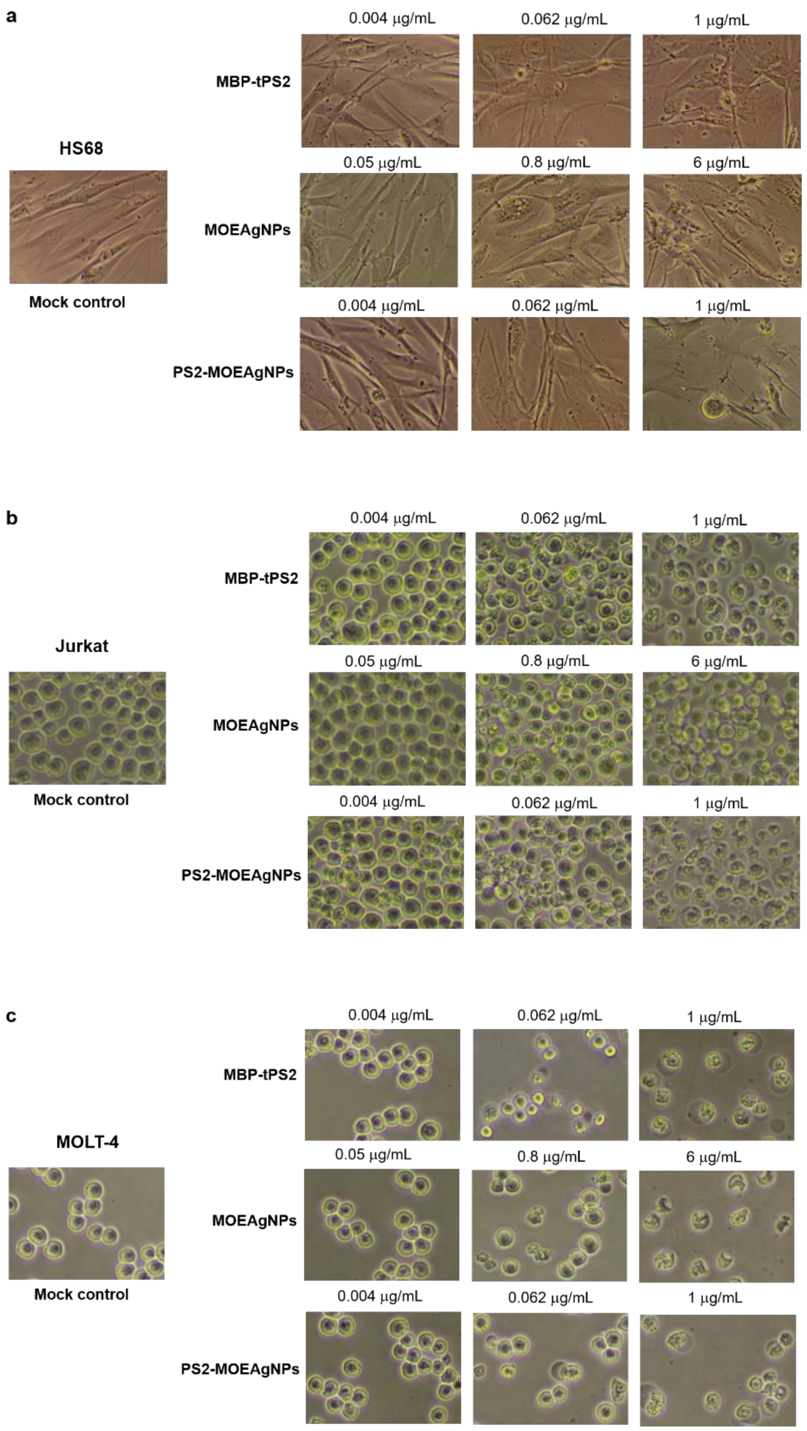

Supplementary Figure S2. Morphology of Hs68, Jurkat, and MOLT-4 cells after treated with MBP-tPS2, MOEAgNPs, and PS2-MOEAgNPs. Hs68 (a), Jurkat (b), and MOLT-4 (c) cells were treated with MBP-tPS2, MOEAgNPs, and PS2-MOEAgNPs at different concentrations for 24 h and then viewed by an inverted light microscope (magnified 10×). PBS buffer was used as a mock control.
